# A single gene mutation predicts response to immune checkpoint blockade in ovarian clear cell carcinoma

**DOI:** 10.1002/1878-0261.70104

**Published:** 2025-07-30

**Authors:** Matheus Henrique Dias, René Bernards

**Affiliations:** ^1^ Division of Molecular Carcinogenesis Oncode Institute, The Netherlands Cancer Institute Amsterdam The Netherlands; ^2^ Princess Máxima Center for Pediatric Oncology Utrecht The Netherlands

**Keywords:** biomarker, Immune checkpoint blockade, ovarian cancer, Protein Phosphatase 2A

## Abstract

There is a lack of genetic biomarkers for predicting response to immune checkpoint blockade (ICB) therapy in cancer. The recent discovery that loss‐of‐function mutations in the gene encoding the protein phosphatase 2A (PP2A) scaffold protein PPP2R1A confer sensitivity to immune checkpoint blockade in ovarian clear cell carcinoma, therefore represents a breakthrough. Mechanistically, mutations in the *PPP2R1A* gene induce a strong interferon gamma response in tumor cells, which enhances infiltration of activated CD8+ T cells into the tumor. The activity of these T cells is then fortified by ICB. Furthermore, preclinical studies have shown that PP2A inhibition leads to the generation of neoantigens by disrupting RNA splicing, and PP2A inhibition can remodel the immune microenvironment of tumors to enhance responses to ICB. The finding that loss‐of‐function *PPP2R1A* mutations predict benefit from immunotherapy also suggests that pharmacological inhibition of PP2A may act synergistically with ICB therapy.

## Graphical abstract

Loss‐of‐function mutations in the scaffold subunit of protein phosphatase 2A (PP2A), PPP2R1A, sensitize ovarian clear cell carcinoma to immune checkpoint blockade (ICB) by enhancing the interferon gamma response and promoting antitumor immune cell infiltration. Pharmacological inhibition of PP2A may replicate these effects, triggering both cancer cell‐intrinsic and ‐extrinsic changes that sensitize PP2A wild‐type tumors to ICB.

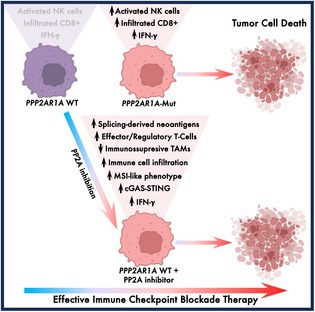

**Abbreviations**
BRAF: V‐Raf Murine Sarcoma Viral Oncogene Homolog B
CD8: cluster of differentiation 8
CTLA4: Cytotoxic T‐lymphocyte antigen 4
EGFR: Epidermal Growth Factor Receptor
ICB: Immune Checkpoint Blockade
IFN‐γ: Interferon gamma
KRAS: Kirsten rat sarcoma viral oncogene homolog
OCCC: Ovarian Clear Cell Carcinoma
PD1: Programmed Cell Death Protein 1
PDL1: Programmed Cell Death Ligand 1
PP2A: Protein Phosphatase 2A
PPP2R1A: Protein Phosphatase 2 Scaffold Subunit A

## 1 The search for biomarkers of response to immune checkpoint blockade

Cancer chemotherapy is well‐known for its lack of predictive biomarkers that can identify which patients are likely to respond to treatment. The introduction of targeted therapies significantly changed this, as their use is often connected to the presence of a specific gene mutation. This led to the development of the so‐called companion diagnostic biomarker tests that can foretell responses to targeted agents. Examples include the detection of activating *BRAF* gene mutations, which predict response to selective BRAF inhibitors, and genetic tests for certain *EGFR* mutations that predict response to EGFR inhibitors. More recently, these biomarkers have become even more precise, with only patients carrying a specific *KRAS* gene mutation (G12C) being likely to respond to drugs such as adagrasib and sotorasib.

The introduction of cancer immune checkpoint blockade (ICB) agents, such as anti‐CTLA4, anti‐PD1, and anti‐PDL1, in 2011 was widely regarded as a breakthrough in cancer treatment. However, when it came to predicting responses, we were initially thrown back into the dark ages, as predicting who would respond to these therapies proved difficult. Patients with cancers such as melanoma and lung cancer were more likely to respond, but not all patients with these cancers did so. Over time, three clinically useful biomarkers of response to ICB have been identified. First, high tumor mutational burden was linked to better responses [1]. Second, expression of PD‐L1 on the tumor predicted responsiveness to anti‐PD1 and anti‐PD‐L1 [2]. Third, microsatellite‐instable (MSI) tumors showed a higher response rate to immunotherapy [3]. As a result, MSI status became the first tissue‐agnostic biomarker approved by the Food and Drug Administration in 2017. The MSI phenotype is complex and is usually determined by testing for the absence of one of four mismatch repair proteins: MLH1, PMS2, MSH2, and MSH6. However, until now, a direct link between a mutation in a single gene and response to ICB, as seen with targeted therapies, has remained elusive.

## 2 A gene mutation predictive of immune checkpoint blockade

Dai *et al*. [4] publication marks a significant breakthrough, demonstrating that ovarian clear cell carcinoma (OCCC) patients with mutations in the *PPP2R1A* gene are more likely to respond to ICB. OCCC patients with the *PPP2R1A* mutation had a median overall survival of 66.9 months, compared to only 9.2 months for those without the mutation. *PPP2R1A* encodes the main scaffold subunit of protein phosphatase 2A (PP2A), and inactivating mutations in this gene are known to decrease PP2A's enzymatic activity.

Since response to ICB depends on a multitude of factors, it is *a priori* remarkable that a single gene mutation can be associated with ICB therapy response. This may be explained by the notion that PP2A is a pleiotropically acting enzyme, affecting a range of biological processes. So, how does the loss of PP2A activity increase ICB sensitivity in OCCC? A first mechanism was revealed using RNA sequencing of OCCC tumor samples, which showed that tumors with *PPP2R1A* mutations displayed activation of the interferon gamma (IFN‐γ) response pathway, which is linked to enhanced responses to ICB [5]. Consistently, these mutant tumors exhibited higher infiltration of CD8+ T cells and activated natural killer (NK) cells. In the literature, several additional immune‐related effects have been described resulting from pharmacological inhibition of PP2A. For instance, inhibiting PP2A activity can disrupt mRNA splicing, resulting in the generation of neo‐antigens that are presented on MHC class I molecules [6]. Moreover, PP2A inhibition can cause an MSI‐like phenotype in cancer cells and activate the cGAS/STING pathway, both of which are known to sensitize cells to ICB [7, 8]. Therefore, both cancer cell‐intrinsic and immune microenvironment‐mediated effects may enhance the responsiveness of *PPP2R1A*‐mutant tumors to ICB.

Mutations in *PPP2R1A* occur at a frequency of between 4% and 9% in OCCC but are relatively rare in other cancers (1% mutation rate across cancers in cBioPortal). Is this immune effect of *PPP2R1A* mutation limited to OCCC, or is *PPP2R1A* mutation a biomarker of response across cancer types? To address this, Dai et al. analyzed a diverse cohort of 1661 patients treated with ICB and again found significantly longer overall survival in the *PPP2R1A* mutant group, while no differences in survival were seen in 7564 patients treated with other therapies. This finding rules out a prognostic effect of the mutation on patient outcome. It is therefore likely that the enhancing effect of *PPP2R1A* mutations on ICB is context‐independent.

## 3 Clinical implications

**Fig. 1:**
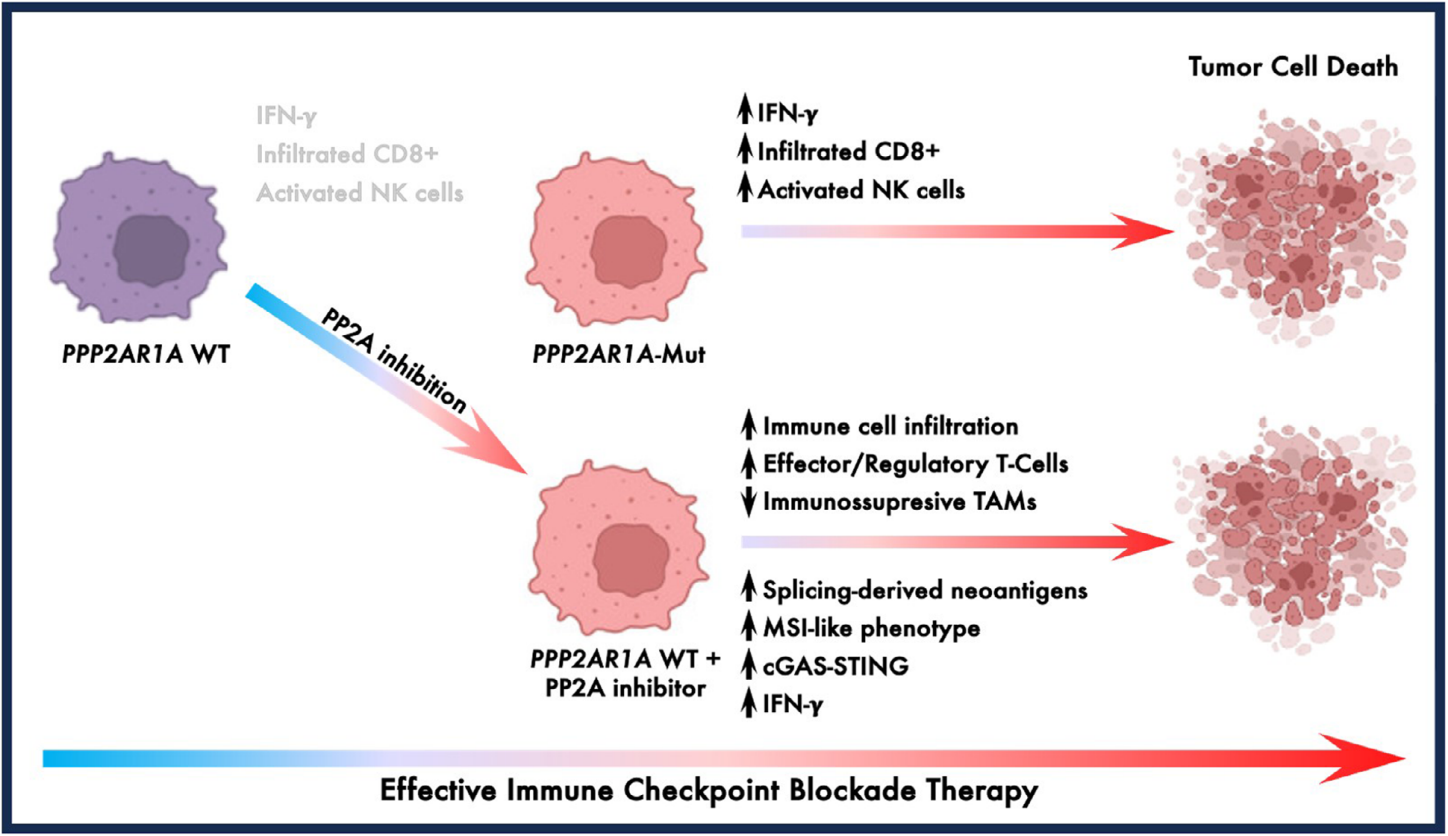
Loss‐of‐function mutations in PPP2R1A enhance the interferon gamma response and promote antitumor immune cell infiltration, sensitizing immunologically “cold” ovarian clear cell carcinoma to immune checkpoint blockade. Pharmacological inhibition of PP2A can mimic these effects across different cancer models by inducing both tumor cell‐intrinsic and extrinsic changes that sensitize PP2A wild‐type tumors to immune checkpoint blockade.

While the frequency of *PP2R1A* mutations across cancer types is low, the total number of patients having *PPP2R1A* mutant tumors that could benefit from ICB therapy is significant. Fortunately, the *PPP2R1A* gene is part of the widely used MSK‐Impact gene sequencing panel for precision oncology, enabling the identification of such patients. A second potential implication of the findings of Dai *et al.* is that pharmacological inhibition of PP2A could phenocopy the genetic loss of PPP2R1A function. There is currently one PP2A inhibitor in clinical development, LB‐100, and this molecule indeed shows synergy with ICB in multiple preclinical models (reviewed in [9]). In the case of systemic inhibition of PP2A with small‐molecule drugs, there is a potential additional positive effect, as PP2A inhibition has been shown to enhance CD8+ T‐cell proliferation and activation, while reducing the number of immunosuppressive macrophages within the tumor microenvironment [9, 10]. Thus, pharmacological inhibition of PP2A may act more broadly on the immune cell compartment than genetic inactivation of PP2A in the tumor through mutation of *PPP2R1A* (Fig. 1). The lack of context dependency of the effect of *PPP2R1A* mutations on ICB responses also suggests that pharmacologic inhibition of PP2A may enhance ICB responses across a range of tumors. Consistent with this, inhibition of PP2A also provoked a strong IFN‐γ response in colon cancer cells [11]. There are currently two ongoing clinical trials to test the combination of the PP2A inhibitor LB‐100 with ICB, one in ovarian clear cell cancer and one in colorectal cancer (NCT06065462 and NCT06012734). There are many ongoing clinical trials combining a range of drugs with ICB. While the outcome of these studies is uncertain, the rationale for combining PP2A inhibition with ICB just became a lot stronger.

## Conflict of interest

R.B. is a member of the board of directors of Lixte Biotechnology Holdings and received research funding from this company.

## Author contributions

M.H.D. and R.B conceived of and wrote this article.
